# Supplementary material for: Absence of critical thickness for polar skyrmions with breaking the Kittel’s law
Source: Nat Commun. 2023 Jun 8;14:3376. doi: 10.1038/s41467-023-39169-y (PMC10250330; doi:10.1038/s41467-023-39169-y)
Supplement: Supplementary file 1 — Supplementary Information [file 41467_2023_39169_MOESM1_ESM.pdf]

Supplementary Information for:

Absence of critical thickness for polar skyrmions with breaking the Kittel's law

Feng-Hui Gong[1,2,†], Yun-Long Tang[1,†], Yu-Jia Wang[1,†], Yu-Ting Chen[1,2,†], Bo Wu[3], Li-Xin Yang[1], Yin-Lian Zhu[1,3*] & Xiu-Liang Ma[1,3,4*]

[1]Shenyang National Laboratory for Materials Science, Institute of Metal Research, Chinese Academy of Sciences, Wenhua Road 72, Shenyang 110016, China.

[2]School of Materials Science and Engineering, University of Science and Technology of China, Wenhua Road 72, Shenyang 110016, China.

[3]Bay Area Center for Electron Microscopy, Songshan Lake Materials Laboratory, Dongguan 523808, Guangdong, China.

[4]Institute of Physics, Chinese Academy of Sciences, Beijing 100190, China.

[†]These authors contributed equally: Feng-Hui Gong, Yun-Long Tang, Yu-Jia Wang, Yu-Ting Chen.

[*]e-mail: ylzhu@imr.ac.cn; xlma@iphy.ac.cn

This PDF file includes:

Supplementary Notes 1 to 4

Supplementary Figs. 1 to 22

Supplementary Table 1 and 2

Supplementary Note 1

Here, we will systematically discuss changes in the shape of satellite peaks in XRD patterns. Previous results also show that the shape of the vortex satellite peaks may be slight differences (the extended satellite peaks for the 16/16 superlattices[29] and the circular satellite peaks for the 20/20 superlattices[20]). However, these satellite peaks all represent vortex structures. In addition, the SAED patterns also show different satellite diffraction spots[18,20]. In fact, some experimental factors may also affect the shape of satellite peaks, such as diffraction geometry, specific X-ray optical components, the setup of each measurement, and the determination of the detection system. In our work, in addition to the information of reciprocal space, a large number of TEM images were analyzed in detail to study the microstructures of the superlattices, therefore, the shape of the satellite peaks may not directly affect our discussion of the scaling law.

We also make some other considerations about the possible reasons for the different shapes of skyrmions satellite peaks. The elongated satellite peaks of the thicker superlattice (n = 17 ~ 39 u.c.) may be caused by the inhomogeneous distribution and morphology of skyrmions, which was further confirmed by under-focus HAADF-STEM images (supplementary Figs. 9, 11). And the circular satellite peaks imply that more ordered and regular skyrmions exist in the PTO_{12}/STO_{12} superlattices, which was also further confirmed by under-focus HAADF-STEM images (supplementary Figs. 10, 11).

Supplementary Note 2

Kittel's law has a wide range of applications, but also has limitations. The derived Kittel's law is based on two basic assumptions. On the one hand, the domain wall thickness is negligible compared to the domain size. For example, the 180 °domain wall is a planar with 1 ~ 2 u.c. thickness, and the 180 °domain wall thickness is negligible. However, in our work, the polar skyrmions observed in the cross-sectional are similar to the 180 °domain. But in the planar-view, the polar skyrmion is significantly different from the 180 ° domain. Although topological skyrmions have complex polarization configurations, they appear as cylinders or spheres as a whole. The skyrmion domain walls became so broad that they could not be ignored. It is very difficult to determine precisely the domain wall region in the case of skyrmions or vortices. On the other hand, the stray field lines connecting one domain to its neighbors are much denser than the field lines connecting one face of the domain to the opposite one. That is to say, the period of the domain is much less than the film thickness. However, in our work, when the superlattices thickness is lower than a certain level, the period of polar domain does not decrease but rises. As a result, the period of polar domain is obviously greater than the film thickness, where the electrostatic interaction with the opposite surface starts to take over. It seems to violate the original assumption that the period of the domain is much less than the film thickness.

In a word, it is reasonable that the period of polar skyrmions and ultrathin film thickness do not conform to Kittel's law.

Supplementary Note 3

We agree that periodic *a*/*c*, a_1/a_2 or vortex domains in the PTO/STO superlattices may show similar RSM super-modulations. But this is not the case in our work. We have several considerations below:

i: If a_1/a_2 domains are present in the superlattices, the Bragg peaks and satellite peaks of a_1/a_2 domains should appear at the positions q_x = 7.7 nm^{-1} in RSM (Fig. 1). The a_1 and a_2 domains are equivalent in-plane, so there will be two symmetrical diffraction spots in close proximity at Bragg peak. However, neither the a_1/a_2 Bragg peaks nor the a_1/a_2 satellite peaks were found in all RSM. Similarly, if periodic *a*/*c* domains are present in the superlattices, the Bragg diffraction spots of the *c* domains will appear at the positions q_x = 7.2 nm^{-1} in RSM (Fig. 1). However, no Bragg diffraction spots of *c* domains can be identified. We then speculate that no *a*/*c* or a_1/a_2 domains form in $(PTO_n/STO_n)_{10}$ superlattices grown on the STO substrates. In addition, strain analysis shows that *a*/*c* domains and a_1/a_2 domains hardly appear in the superlattices grown on the STO substrates (Supplementary Table 2).

ii: *a*/*c*, a_1/a_2, vortex and flux-closure are ferroelastic domains modulated by tensile strain, whereas *c*/*c* 180° domain and skyrmion are ferroelectric domains significantly affected by electric boundaries. Strain analysis helps us understand that *a*/*c* and a_1/a_2 domains unlikely exist in the PTO/STO superlattices grown on the STO substrates (Supplementary Table 2) since the lattice mismatch between STO and PTO is almost zero. Next, when the PTO/STO superlattices were grown on the scandate substrates, a periodic vortex array maybe formed in the PTO/STO superlattices as reported previously. However, due to the effect of strain, it is possible that vortices and ferroelastic domains (*a*/*c*, a_1/a_2) coexist in the PTO/STO superlattices grown on the scandate substrates such as $DyScO_3$, which gives large tensile strains to the PTO/STO superlattices[29]. Moreover, flux-closures and vortices may be essentially the same topological structure (on $GdScO_3$ substrate), and reducing the thickness of the superlattices can result in the transition from flux-closures to vortices[15,18,22]. Thus in summary here, the 180° domains are possibly formed in the epitaxial single PTO films grown on the STO substrates, but the *a*/*c* and a_1/a_2 domains, and vortex topology are unlikely from the strain aspect.

iii: Most importantly, we have further used TEM and STEM based methods, up to the atomic scale level, to reveal the structures of the skyrmions here (Figs. 1,3 and supplementary Figs. 5,6,9-11,15,16). The *c*/*c* 180 °domain walls are sharp, and the *c*/*c* 180 ° domains are regular periodic arrangement[58]. The diffraction contrast images showed that the domain walls are fuzzy and do not show a regular periodic arrangement (Fig. 1 and supplementary Fig. 5). The diffraction contrast image shows that the domains are short-range order, as previously reported. We observed the 180 °domain along the [001] direction, which is distributed in periodic strips. But the under-focus images of the planar-view shows that it has a circular character (Fig. 3, Supplementary Figs. 9-11,15,16). Polarization mapping also accords with the skyrmions polarization characteristics (Fig. 3, Supplementary Figs. 9,10,15,16). The diffraction contrast images and polarization images are consistent with the skyrmions characteristics, and it is confirmed that the domain is not *c*/*c* 180 °. Moreover, in the planar-view, the a_1/a_2 domain walls are sharp along the [110] or [$1\bar{1}0$], but we observed the circular domains in our work (Fig. 3, Supplementary Figs. 9-11,15,16). Therefore, what we observed in the PTO/STO superlattices grown on the STO substrates is not consistent with the *a*/*c* and a_1/a_2 domain signature. In addition, in the planar-view, vortex and flux-closure are also tubular, periodic distributions[18]. But we did not observe vortex with periodic tubular. Polarization analysis shows that they do not conform to the vortex characters. By the way, why the signal to noise is very poor in the RSM here in our case, is because that the periods of these skyrmions are not so perfect as those from either the vortex or a_1/a_2 domains.

In summary, it is unlikely that there are *c*/*c*, *a*/*c*, a_1/a_2 and vortex in PTO layers based on RSM results. Strain analysis showed that *c*/*c*, *a*/*c*, a_1/a_2 and vortex were unlikely to form in PTO/STO superlattices grown on the STO substrates. In addition, TEM images do not reveal the characteristics of *c*/*c*, *a*/*c*, a_1/a_2 and vortex. As a result, no complex mixed phases exist in PTO/STO superlattices grown on the STO substrates. The skyrmions and their evolutions were thus collaborated and convinced by our TEM/STEM and other characterizations.

Supplementary Note 4

Based on the above strain analysis ii and Supplementary Table 2, the *a*/*c*, a_1/a_2 are ferroelastic domain, the vortex and flux-closure suffer from large strain modulation. The formation and transformation of vortex and flux-closure to *a*/*c* and a_1/a_2 domains are affected by strain. *a*/*c*, a_1/a_2 are correlated with vortex, flux-closure. However, *c*/*c* is ferroelectric domain, and the ferroelectric domain is significantly affected by the electric boundary conditions. The skyrmions are also significantly affected by short-circuit conditions (Fig. 4d, Supplementary Figs. 20,21). So the formation of the skyrmion is correlated with the *c*/*c* domain. The effect of strain on skyrmion is relatively weak, which has been confirmed by the phase-field simulation (Fig. 2, Supplementary Figs. 7,8). It is very important for the PTO/STO superlattices to grow on which substrate[18,37,62]. This determines which strain (tensile strain, zero strain, compression strain) the PTO/STO superlattices is modulated by, thus determining the formation of ferroelastic or ferroelectric domains in PTO films.

Supplementary Figures

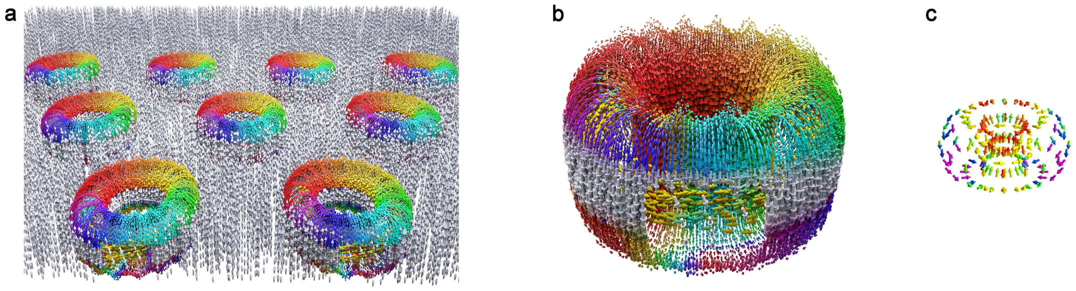

Supplementary Fig. 1. Skyrmions structure. a-c Skyrmions array and skyrmions structure of different size. The arrows represent the **Ps**.

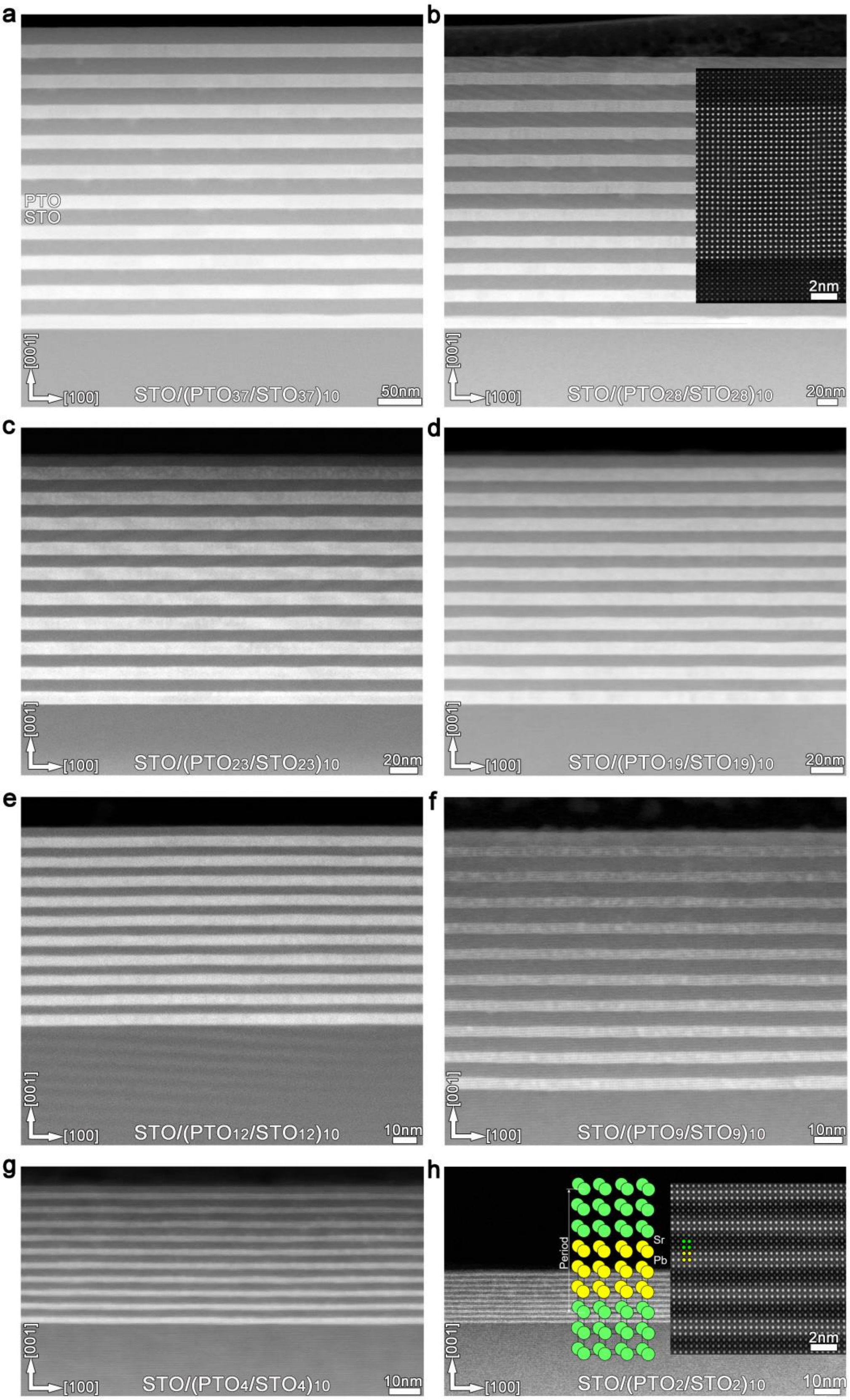

Supplementary Fig. 2. Structural characterization of the (PTO_n/STO_n)$_{10}$ superlattices. a-h Low-magnification STEM images of the cross-sectional of (PTO_n/STO_n)$_{10}$ (n = 37, 28, 23, 19, 12, 9, 4, 2 u.c.) superlattices. The insets in **b** and **h** are local magnified images, respectively.

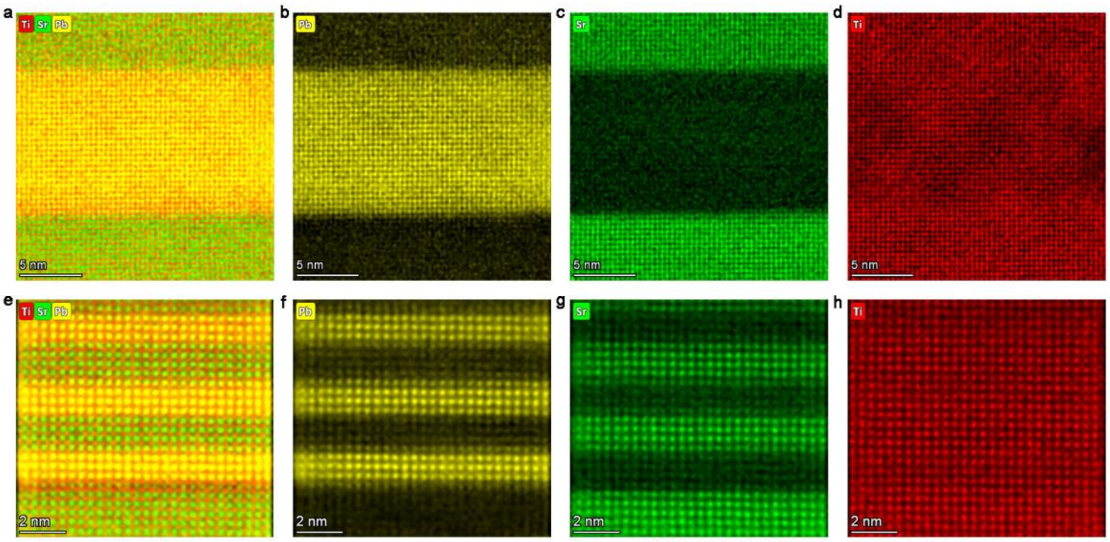

Supplementary Fig. 3. Super-EDS mapping of Sr, Ti and Pb. a-d EDS mapping of the $(PTO_{28}/STO_{28})_{10}$ superlattices. **e-h** EDS mapping of the $(PTO_2/STO_2)_{10}$ superlattices.

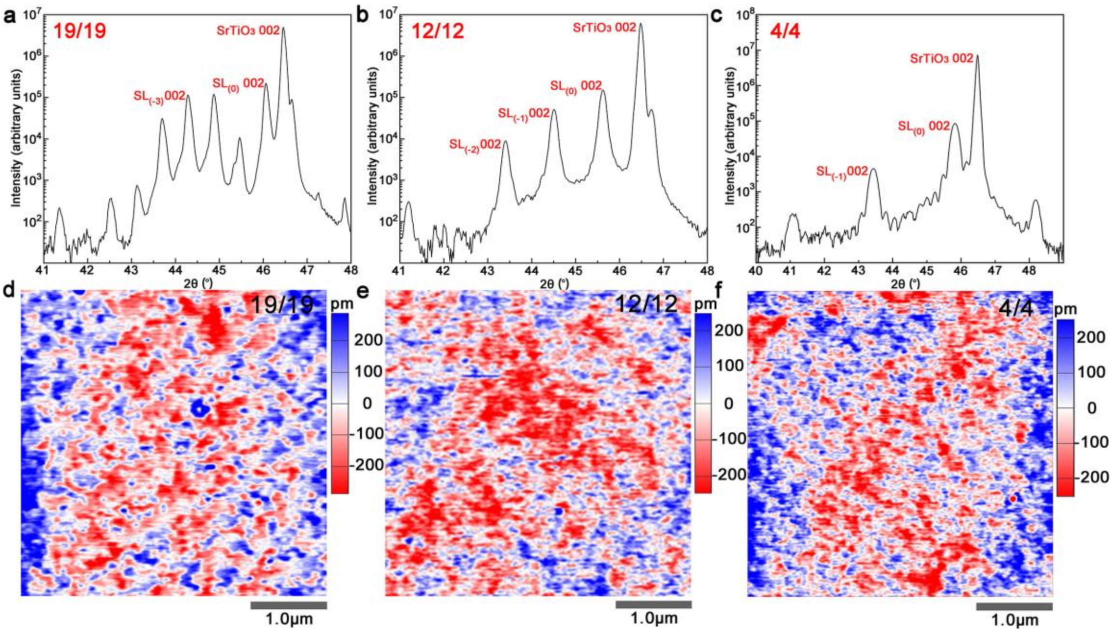

Supplementary Fig. 4. X-ray diffraction and AFM tomography analysis. a-c High-resolution θ-2θ symmetric scans for $(PTO_n/STO_n)_{10}$ (n = 19, 12, 4 u.c.) superlattices. These results reveal that the superlattices have smooth surface and good epitaxy. **d-f** The superlattices surface are very smooth within a unit cell fluctuation.

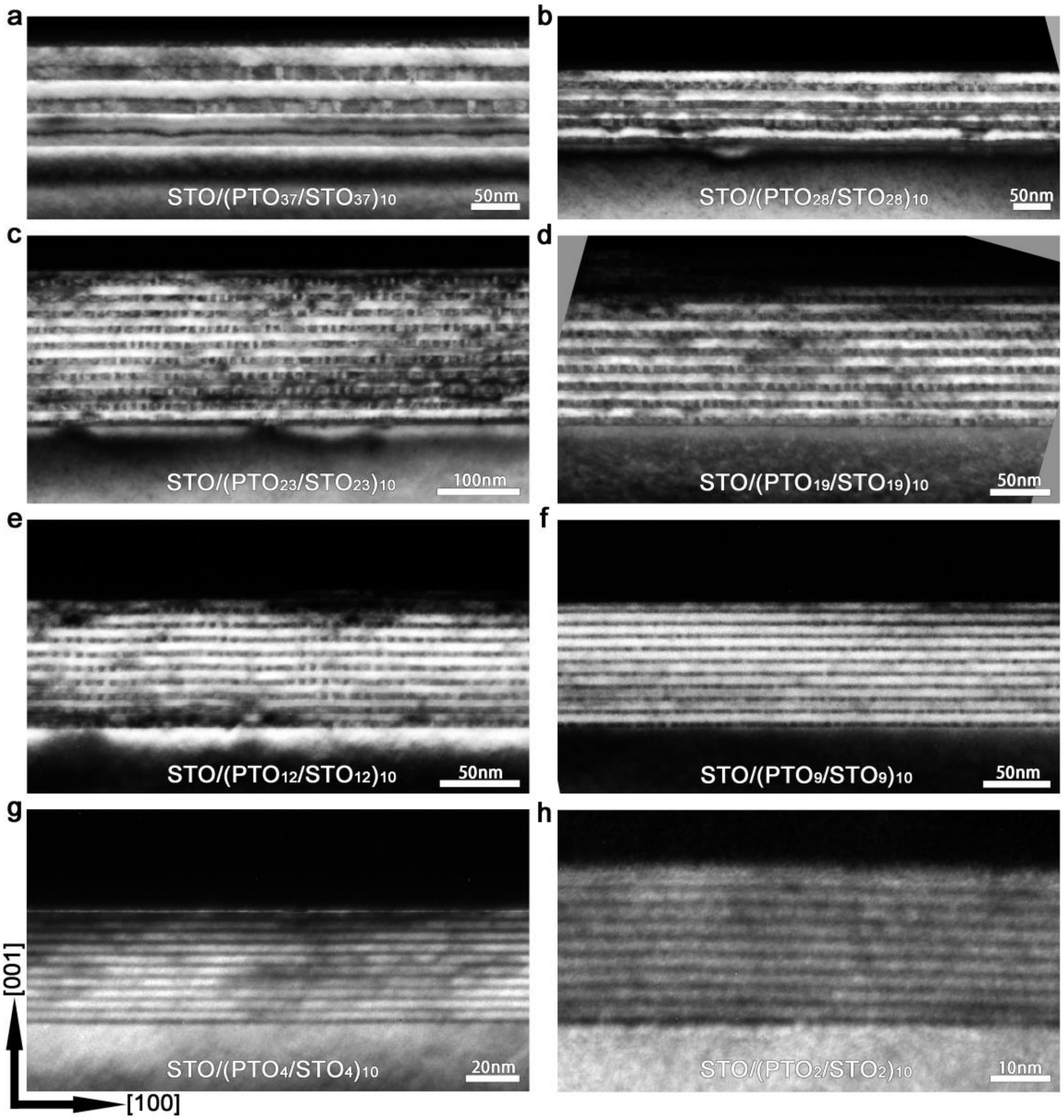

Supplementary Fig. 5. Other areas diffraction contrast images. a-h A range of cross-sectional DF images shows thickness-dependent skyrmions evolution in the $(PTO_n/STO_n)_{10}$ (n = 37, 28, 23, 19, 12, 9, 4, 2 u.c.) superlattices.

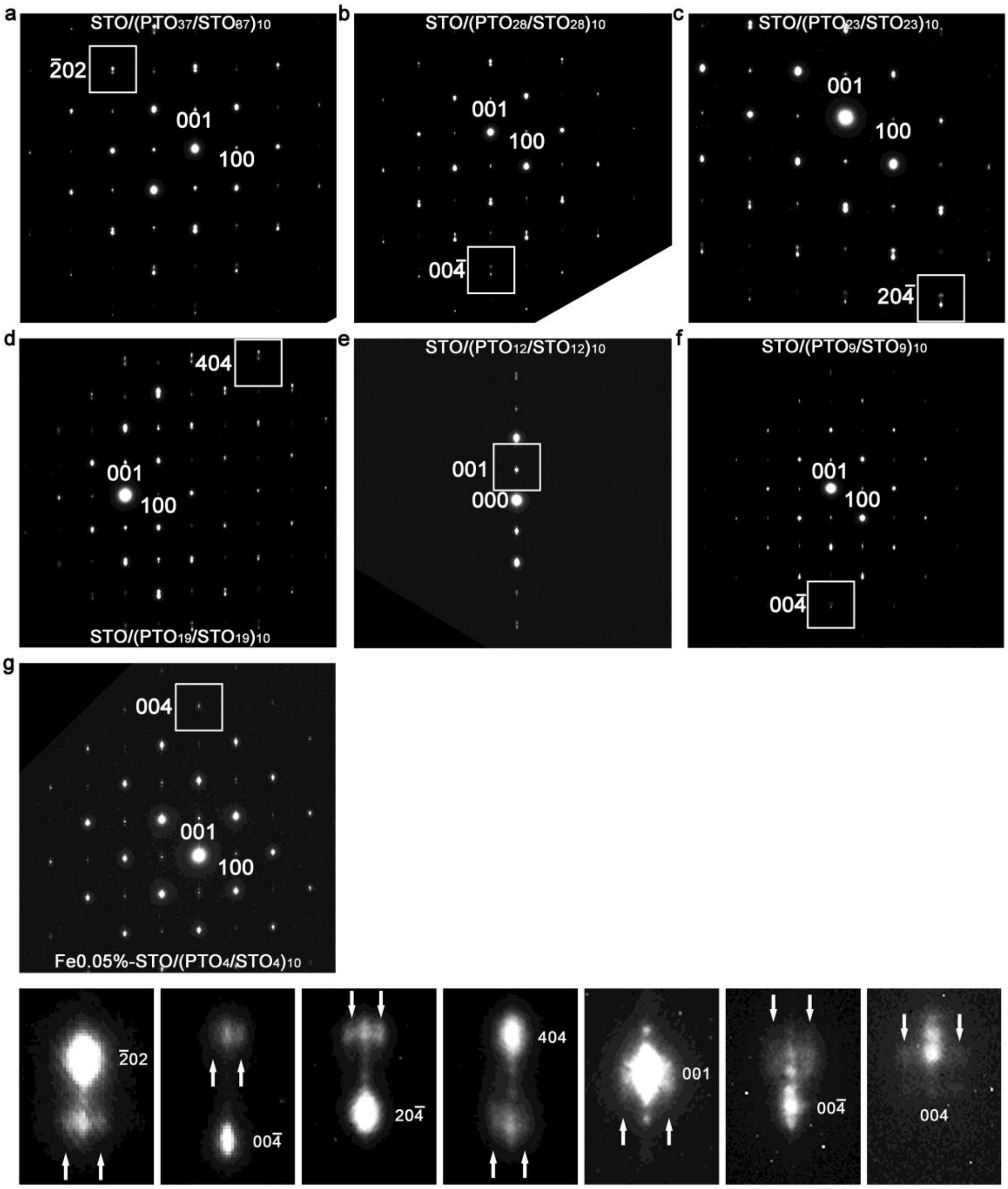

Supplementary Fig. 6. SAED patterns. a-g SAED patterns of the $(PTO_n/STO_n)_{10}$ (n = 37, 28, 23, 19, 12, 9, 4 u.c.) superlattices. Note that **e** is the SAED under the approximate two-beam condition. All the SAED patterns taken from the area including the substrate and superlattice. These enlarged images of single diffraction spot (corresponding to white box) are located in the main text **Fig. 1**.

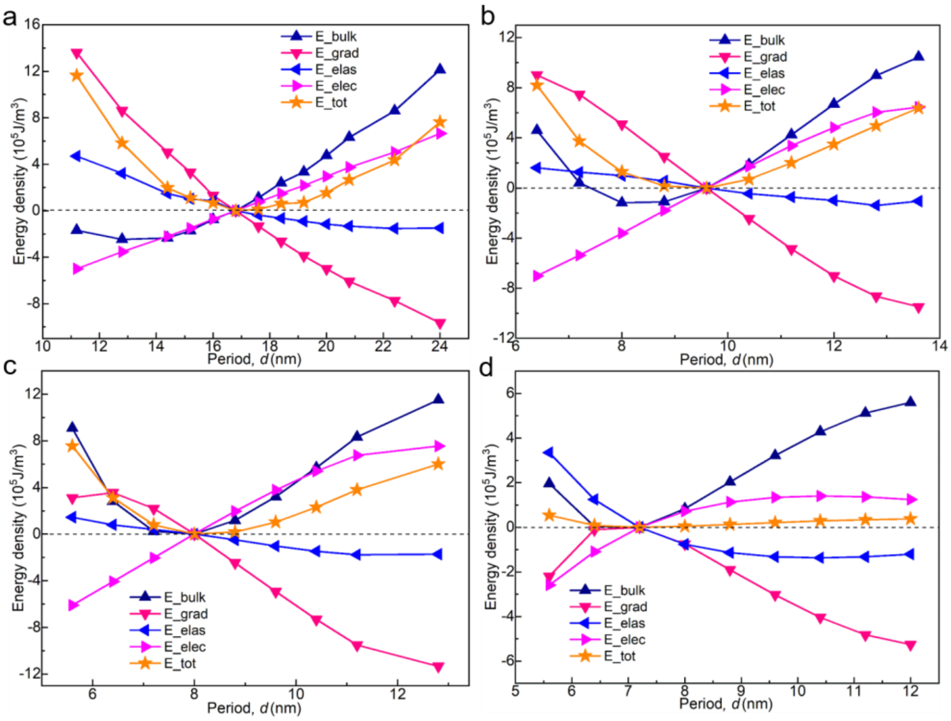

Supplementary Fig. 7. The variance of energy components of the skyrmion lattice for the STO/PTO/STO trilayer with different PTO layer thicknesses. a 12 nm; **b** 4 nm; **c** 3.2 nm; **d** 1.6 nm. The energy components at the optimal periods were taken as the references.

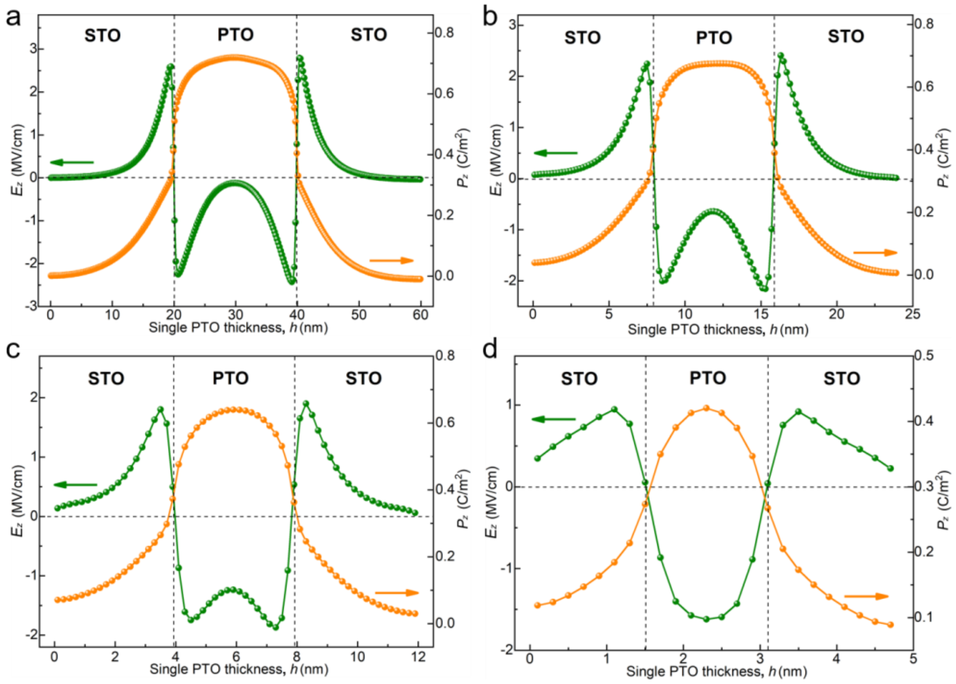

Supplementary Fig. 8. The planar-averaged electric field and polarization profiles along the film normal for the STO/PTO/STO trilayers with different thicknesses. a 20 nm; **b** 8 nm; **c** 4 nm; **d** 1.6 nm.

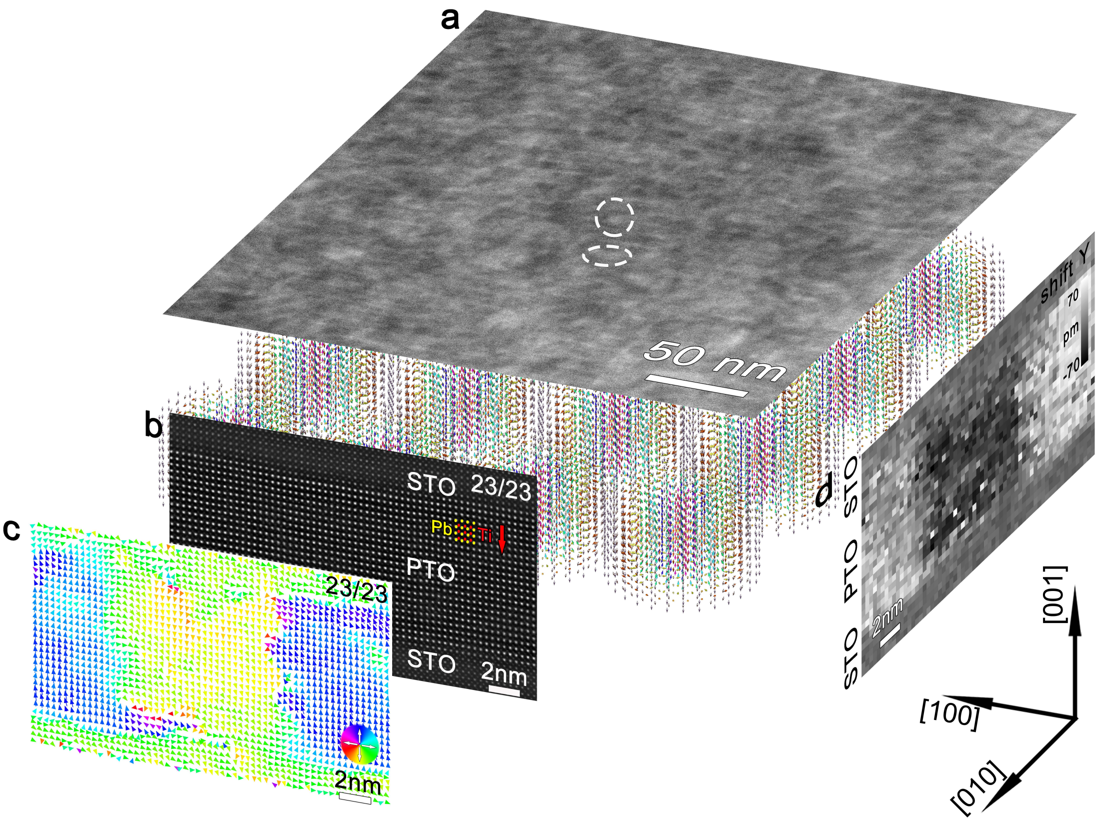

Supplementary Fig. 9. Thickness-dependent 3D polar skyrmions evolution. a Planar-view DF under-focus STEM imaging of $(PTO_{23}/STO_{23})_{10}$ superlattices, showing the widespread occurrence of rounded and elongated polar skyrmions. The individual skyrmion is marked by white dotted circle or ellipse. The 3D skyrmion maps are located under the under-focus STEM images. **b** Atomically resolved cross-sectional HAADF-STEM images of $(PTO_{23}/STO_{23})_{10}$ superlattices. The yellow and red circles denote the Pb and Ti atom columns, respectively, and the red arrow denotes the direction of Ti-displacement (δ_{Ti}) vectors. **c** The reversed δ_{Ti} vector maps (**Ps**), revealing the thickness-dependent skyrmion shrinking process. **d** The shift Y along the out-of-plane direction.

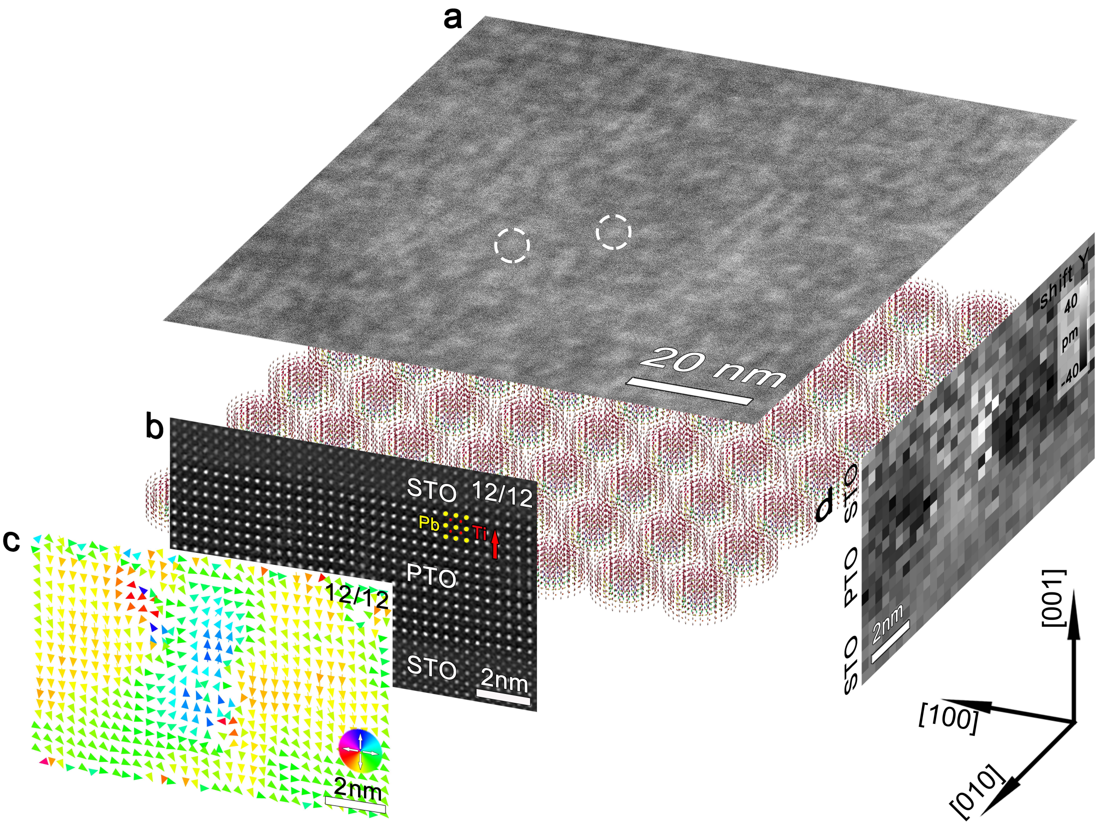

Supplementary Fig. 10. Thickness-dependent 3D polar skyrmions evolution. a Planar-view DF under-focus STEM imaging of $(PTO_{12}/STO_{12})_{10}$ superlattices, showing the widespread occurrence of rounded and elongated polar skyrmions. The individual skyrmion is marked by white dotted circle. The 3D skyrmion maps are located under the under-focus STEM images. **b** Atomically resolved cross-sectional HAADF-STEM images of $(PTO_{12}/STO_{12})_{10}$ superlattices. The yellow and red circles denote the Pb and Ti atom columns, respectively, and the red arrow denotes the direction of Ti-displacement (δ_{Ti}) vectors. **c** The reversed δ_{Ti} vector maps (**Ps**), revealing the thickness-dependent skyrmion shrinking process. **d** The shift *Y* along the out-of-plane direction.

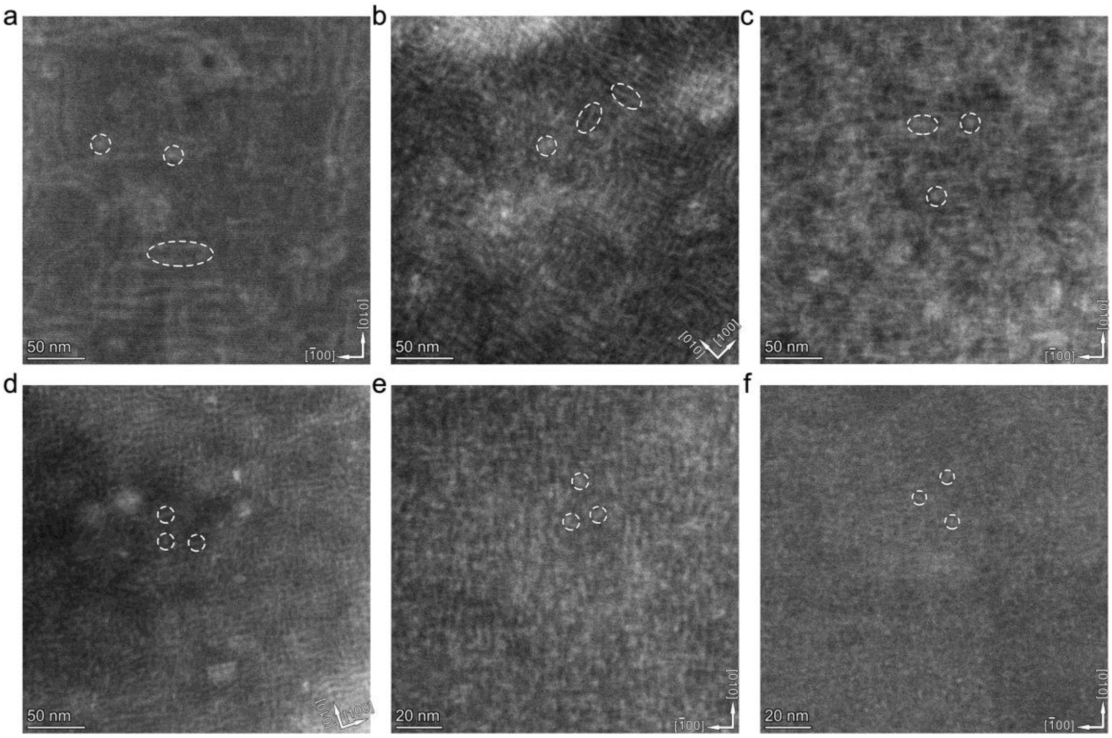

Supplementary Fig. 11. Under-focus STEM imaging of polar skyrmion of the ($PTO_n/STO_n)_{10}$ (*n* = 37, 28, 23, 19, 12, 4 u.c.) superlattices. a-f Thickness-dependent polar skyrmions evolution in PTO/STO superlattices. The individual skyrmion is marked by white dotted circle or ellipse.

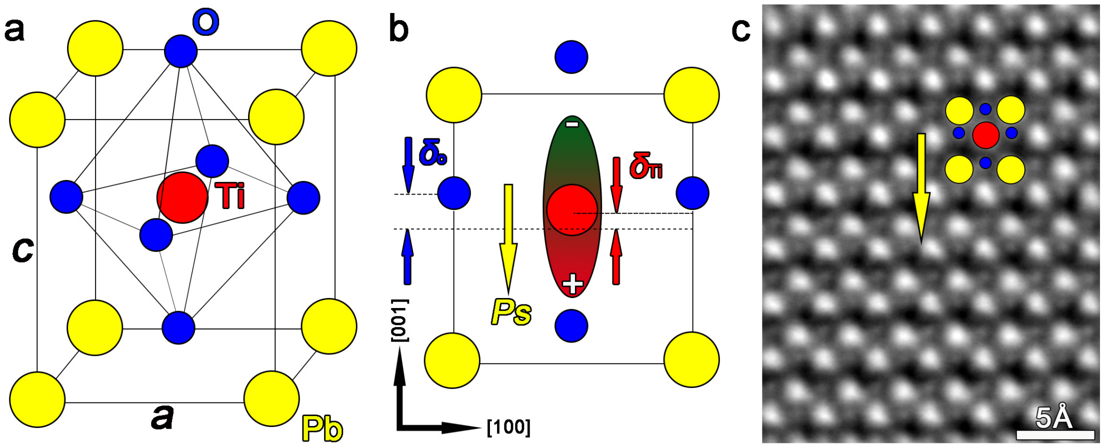

Supplementary Fig. 12. Projection of the PTO unit cell along the [010] direction. a, b Yellow, red and blue circles represent the Pb, Ti and O atoms, respectively. The yellow arrow denotes the direction of **Ps**, which is opposite to the direction of Ti^{4+} displacement direction. Usually, [100] and [010] directions are defined as in-plane directions and [001] directions is defined as out-of-plane directions. **c** The integrated differential phase contrast (IDPC) image of PTO layers in $(PTO_{37}/STO_{37})_{10}$ superlattices, which shows that the direction of δ_{Ti} is the same as the direction of δ_O.

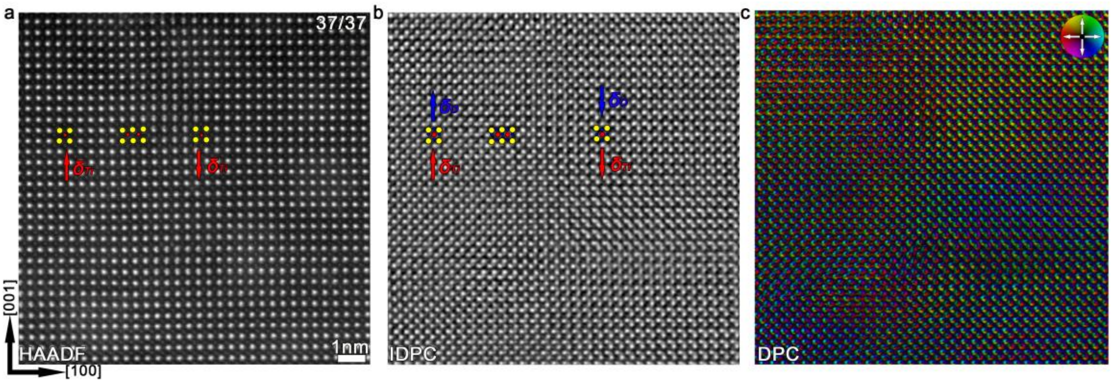

Supplementary Fig. 13. Polarization analysis of ferroelectric skyrmion. a The HAADF-STEM image in the $(PTO_{37}/STO_{37})_{10}$ superlattices. The yellow and red circles denote the Pb nd Ti atom columns, respectively, and the red arrow denotes the direction of Ti-displacement (δ_{Ti}) vectors. **b** The IDPC image corresponding to HAADF-STEM image. The blue circles denote the O atom columns, and the blue arrow denotes the direction of O-displacement (δ_O) vectors. **c** The differential phase contrast (DPC) image, which shows the non-uniform distribution of the electric field.

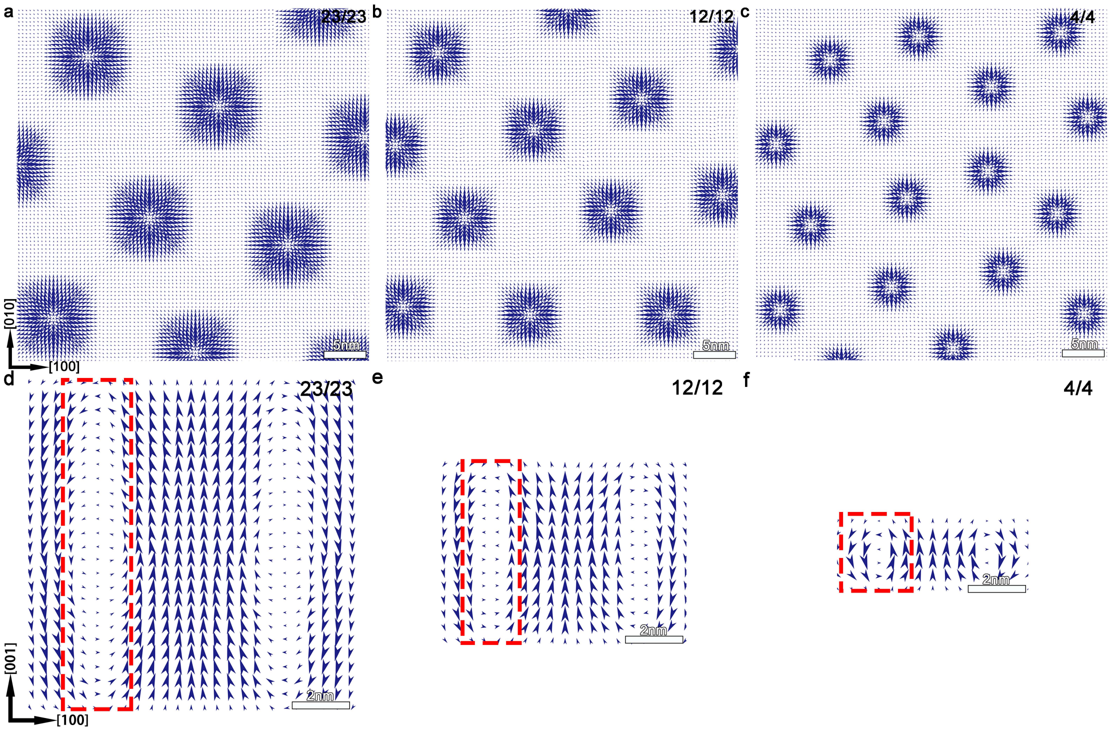

Supplementary Fig. 14. Schematic diagram of polar skyrmions varying with thickness. a-f Evolution of polar skyrmions in PTO/STO superlattices with thickness. The red dotted boxes highlight the evolution of skyrmion structural details.

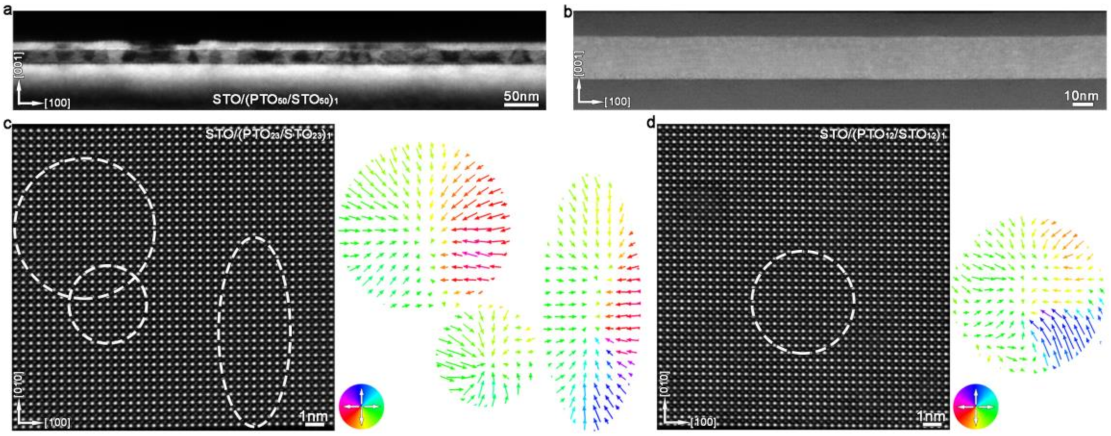

Supplementary Fig. 15. The ferroelectric skyrmions in (PTO_n/STO_n)$_1$ (*n* = 50, 23, 12 u.c.) bilayers. a, b The cross-sectional DF image and low-magnification HAADF-STEM image of (PTO_{50}/STO_{50})$_1$ bilayer. **c, d** The planar-view HAADF-STEM images and extracted skyrmion polarization mappings of (PTO_n/STO_n)$_1$ (*n* = 23, 12 u.c.) bilayers. The white dotted circles and ellipse mark skyrmions areas.

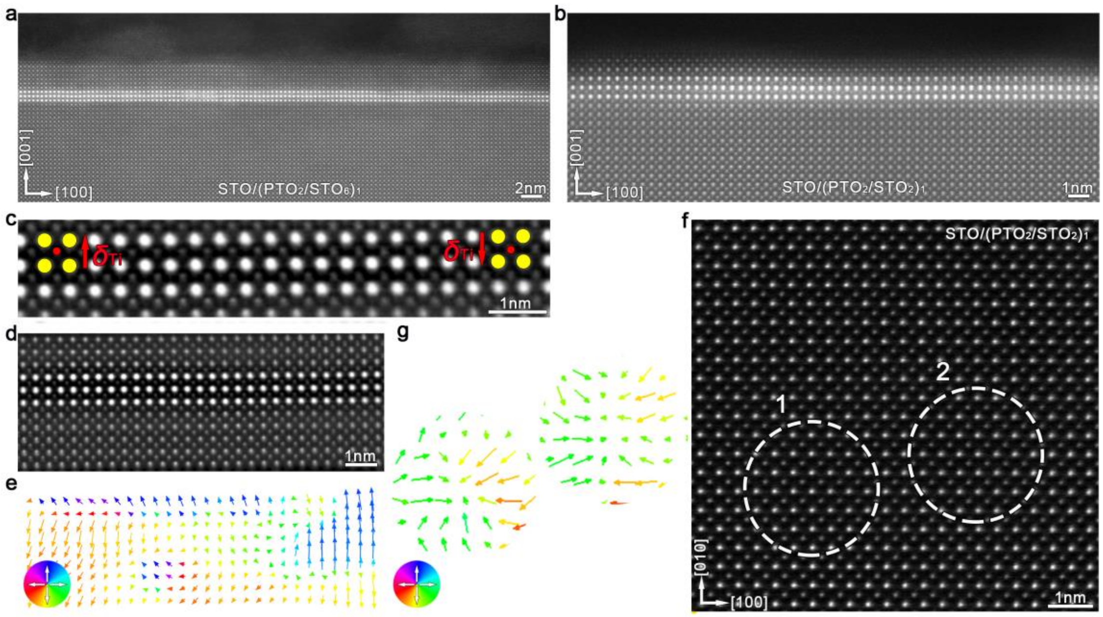

Supplementary Fig. 16. The ferroelectricity and polar skyrmions in the ultrathin bilayers. a, b The HAADF-STEM images of (PTO_2/STO_6)$_1$ and (PTO_2/STO_2)$_1$ bilayers. **c** The HAADF-STEM image shows clear Ti^{4+} displacement. **d, e** The HAADF-STEM image and corresponding polarization map. **f** The planar-view HAADF-STEM image of (PTO_2/STO_2)$_1$ bilayer. **g** Polarization map extracted from the white dotted circles areas in **f**.

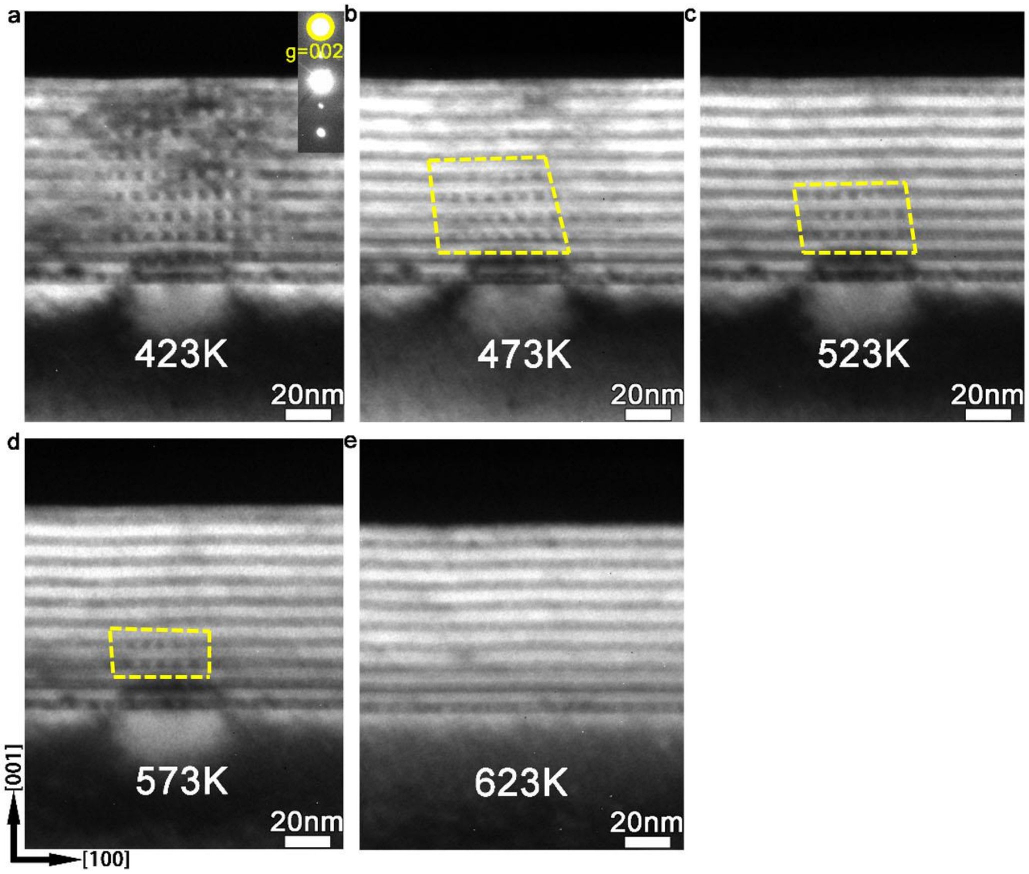

Supplementary Fig. 17. Temperature control of the topological phase transition of the polar skyrmion in the $(PTO_{12}/STO_{12})_{10}$ superlattices. a-e Temperature-dependent DF images from 423 K to 623 K. The inset in **a** is SAED pattern. The polar skyrmions are surrounded by yellow dashed box.

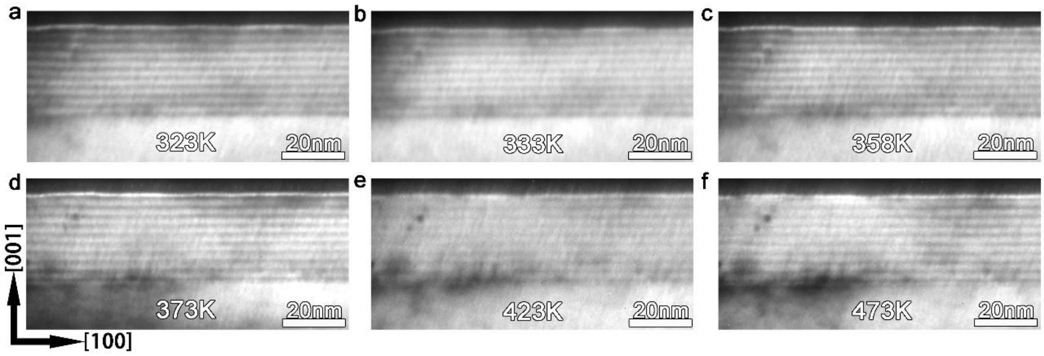

Supplementary Fig. 18. In-situ heating of the $(PTO_2/STO_2)_{10}$ superlattices. a-f Temperature-dependent DF images from 323 K to 473 K. The black dot contrast region should be the polar skyrmions, which disappeared around 423 K (150 ℃).

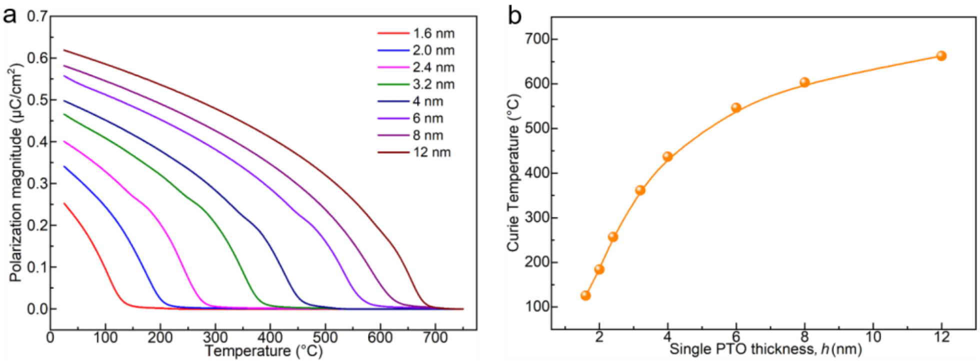

Supplementary Fig. 19. a The evolution of averaged polarization magnitude in the PTO layer with temperature for the STO/PTO/STO trilayers with different thicknesses. **b** The variance of Curie temperature with the film thickness.

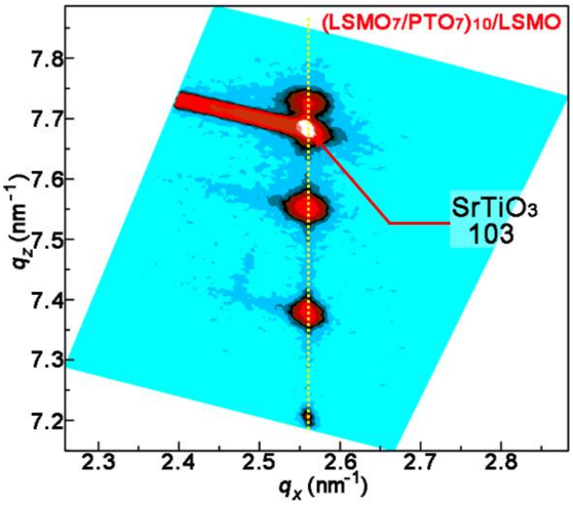

Supplementary Fig. 20. Logarithmic RSM of the $(LSMO_7/PTO_7)_{10}/LSMO$ superlattices. The satellite peak is missing. It shows that there is no polar topological structure.

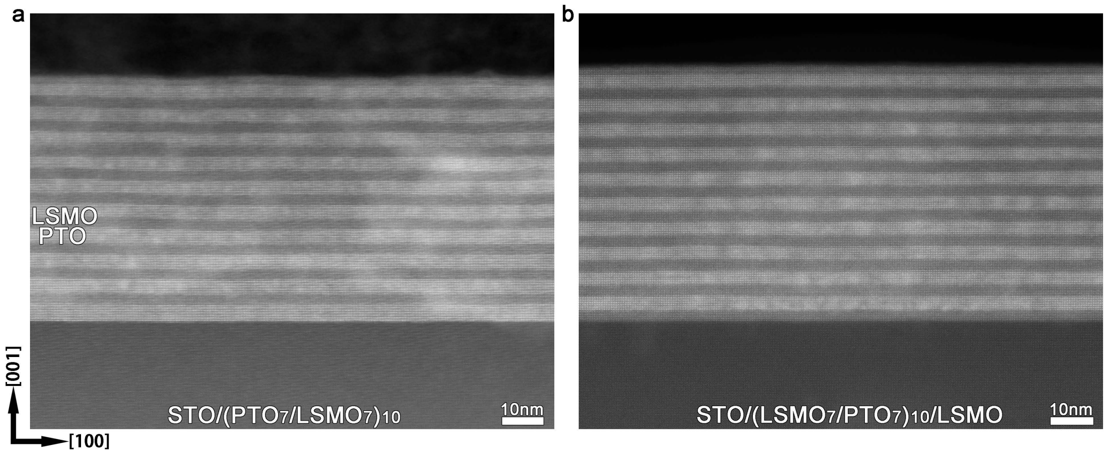

Supplementary Fig. 21. Structural characterization of superlattices. a, b Low-magnification HAADF-STEM images of the $(PTO_7/LSMO_7)_{10}$ and $(LSMO_7/PTO_7)_{10}/LSMO$ superlattices.

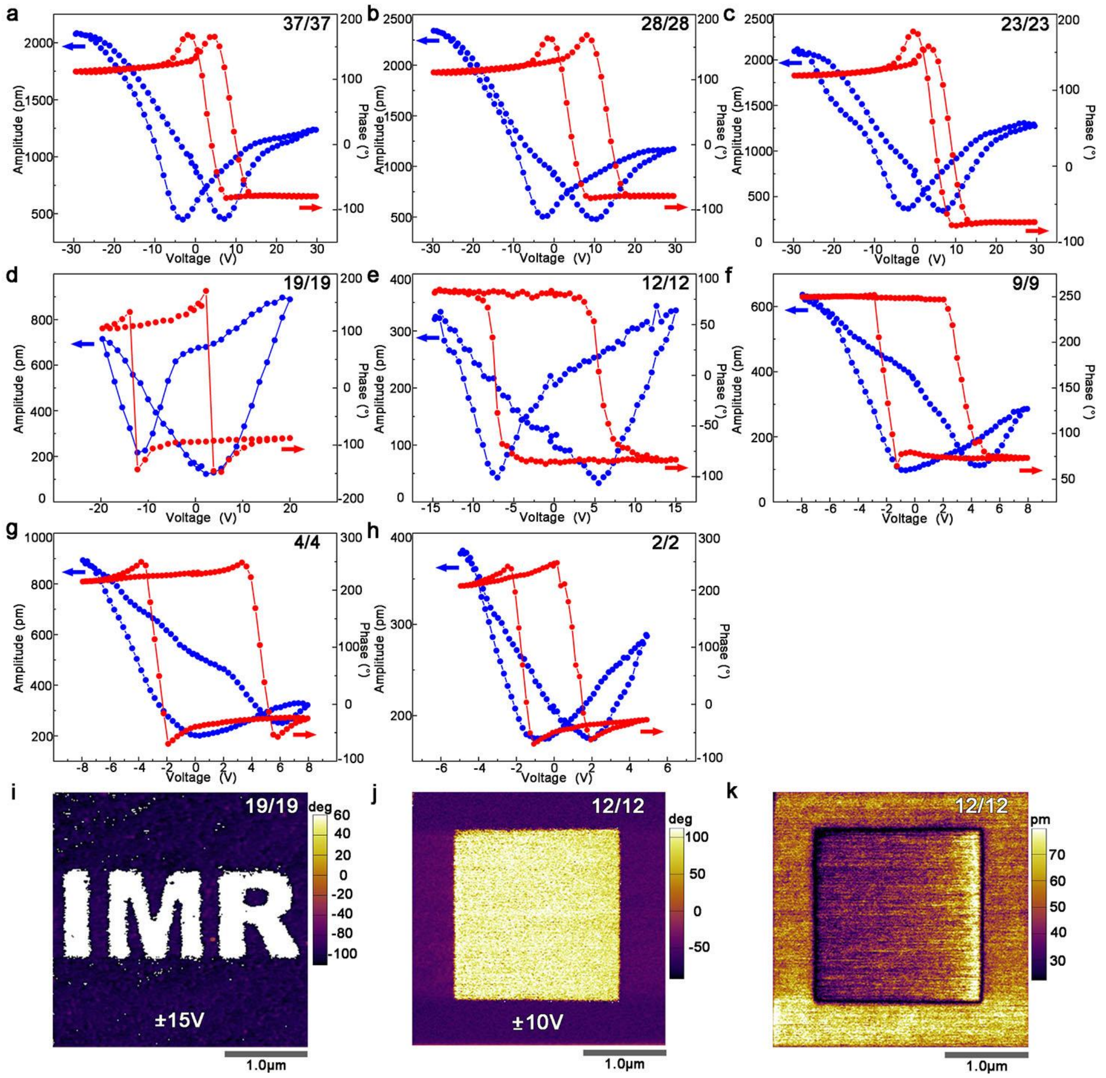

Supplementary Fig. 22. PFM measurement. a-h PFM amplitude–voltage butterfly loop and phase–voltage hysteresis loop of a series of $(PTO_n/STO_n)_{10}$ (n = 37, 28, 23, 19, 12, 9, 4, 2 u.c.) superlattices. **i-k** Domain writing of the $(PTO_n/STO_n)_{10}$ (n = 19, 12 u.c.) superlattices.

Supplementary Table 1 The period-thickness relation obtained by different assumptions and derivations.

Kittel's law	Square root dependence	Annotation	References
$d \propto \sqrt{h}$	$\omega = \sqrt{\frac{\sigma}{U} d}$	ω:width<br>d:thickness	(2)
	$D = \frac{1}{\alpha}\left(\frac{2\gamma H}{\xi G g^2 \ln \alpha}\right)^{\frac{1}{2}}$	D:period<br>H:thickness	(3)
	$W^2 / D = 1.0[\varepsilon_b f(b+g)/b]^{\frac{1}{2}}$	$2W$:period<br>D:thickness	(4)
	$W_{eq} \propto (\frac{h\gamma}{E \in_T^2})^{\frac{1}{2}}$	W:width<br>h:thickness	(5)
	$D^* \approx \left[\frac{16\pi^3 \sigma H}{7\xi(3) G (s_a - s_c)^2}\right]^{\frac{1}{2}}$	D:period<br>H:thickness	(6)
	$l_{\min} = \frac{1}{\omega}\left(\frac{2\pi}{\ln 2}\right)^{\frac{1}{2}}\left(\frac{\gamma h}{G}\right)^{\frac{1}{2}}$<br>$l_{\min} = 2^{\frac{4}{3}} h \exp(\frac{2\pi d}{3\omega^2 h} - \frac{1}{2}) \quad h \ll l$	l:width<br>h:thickness	(7)
	$W_{eq}/2 = \sqrt{h_M h}$	W:width<br>h:thickness	(8)
	$L_{eq} = \sqrt{(2^{n+1}-1)\frac{\sigma_w \pi^3 \varepsilon_0 (1+\sqrt{\kappa_c \kappa_a})h}{8P_s^2 \alpha(n)}}$	$2L_{eq}$:period<br>h:thickness	
	$W \propto \frac{1}{\sqrt{\frac{1}{h} - \frac{h_\delta}{\lambda_D^2}}}$	W:width<br>h:thickness	

Supplementary Table 2 Statistical domain configuration distribution. The difference between superlattices and multilayers is film thickness. Flux-closure is made up of a pair of *c* domains polarized up and down and a pair of *a* domains polarized left and right.

Substrate and strain / Epitaxial film	$SrTiO_3$ (STO) 0	$DyScO_3$ (DSO) 1.28%	$TbScO_3$ (TSO) 1.55%	$GdScO_3$ (GSO) 1.77%	$SmScO_3$ (SSO) 2.26%	$NdScO_3$ (NSO) 2.79%
	Ferroelctric 180 °domain	Ferroelastic a/c a_1/a_2 domain				
Single $PbTiO_3$ film	180 °domain	a/c	a/c	a/c<br>a_1/a_2	a_1/a_2	a_1/a_1
$SrRuO_3/PbTiO_3$ films	Single domain	a/c	a/c	a/c<br>a_1/a_2	a_1/a_2	a_1/a_1
$PbTiO_3/SrTiO_3$ superlattice	skyrmion	vortex	vortex	vortex	vortex	vortex
$PbTiO_3/SrTiO_3$ multilayer		flux-closure	flux-closure	flux-closure	flux-closure	flux-closure
$SrRuO_3/PbTiO_3/SrRuO_3$		vortex				
$La_{0.7}Sr_{0.3}MnO_3/PbTiO_3/$ $La_{0.7}Sr_{0.3}MnO_3$ superlattice	Disappear			vortex		
$SrRuO_3/PbTiO_3/SrRuO_3$ multilayer		flux-closure		flux-closure		
$La_{0.7}Sr_{0.3}MnO_3/PbTiO_3/$ $La_{0.7}Sr_{0.3}MnO_3$ multilayer				flux-closure		
